# Essential therapeutics skills required of junior doctors

**DOI:** 10.1007/s40037-012-0032-1

**Published:** 2012-11-20

**Authors:** Mathew J. Baldwin, Michael Abouyannis, Tehreem F. Butt

**Affiliations:** 1 Ealing Hospital, London, UB1 3HW UK; 2 Department of Clinical Pharmacology, School of Clinical and Experimental Medicine, University of Birmingham, Birmingham, B15 2TT UK; 3 Royal Liverpool University Hospital, Liverpool, UK

**Keywords:** Clinical pharmacology and therapeutics, Junior doctors, Prescribing, Undergraduate medical education

## Abstract

Junior doctors are responsible for the majority of in-hospital prescription errors. Little research has explored their confidence to prescribe, or practical therapeutics related tasks which they are required to perform in day-to-day practice. This survey aimed to explore these areas, gather feedback regarding therapeutics teaching at undergraduate level, and to apply findings to undergraduate training at University of Birmingham. Questionnaire-based survey of all first-year postgraduate doctors (PG1) attending teaching hospitals in the Birmingham and Worcester regions towards the end of the PG1 year. Doctors were asked about difficulties in prescribing, satisfaction with undergraduate training, and how frequently they undertook particular tasks pertaining to therapeutics. Qualitative data on suggestions for improving the curriculum were also collected. Difficulties were commonly encountered with prescribing warfarin, controlled drugs and syringe-driven drugs. Most (87.4 %) had been required to administer intravenous medications. Nearly all had prescribed to ‘special groups’ such as the elderly (100 %) and patients with renal disease (98.3 %). Thirty-seven percent were not satisfied with their undergraduate therapeutics teaching, and many (56.2 %) recommended making teaching more relevant to clinical practice. Many PG1s expressed difficulties in prescribing potentially dangerous medications. Although better than other UK surveys, significant numbers were not satisfied with undergraduate teaching. The strong opinion was for teaching to become more practical and more relevant. Prescriptions which PG1s are commonly asked to write have been described. Findings have guided improvements to undergraduate teaching and assessment in therapeutics at the University of Birmingham, and may offer guidance to other medical schools.

## Introduction

With an expanding national formulary, increased incidence of polypharmacy, and an older patient demographic, the complexities and risks of prescribing have increased. In the UK, incidence of prescribing errors has been reported at 1.5 % for hospital inpatients, and over one quarter of these have been reported as potentially serious [1]. Senior and junior house officers have been found to be responsible for 84 % of prescription errors [1].

The causes of prescription errors are multi-factorial originating from both individual and organizational factors [2]. The quality of undergraduate therapeutics teaching has been reported as a contributor [2, 3]. An Aberdeen-based study reported that 30 % of first-year postgraduate doctors (PG1s) felt that their knowledge of clinical pharmacology and therapeutics was ‘poor’ or ‘very poor’, and only 56 % felt their undergraduate teaching had equipped them to prescribe safely and rationally [4].

Prescribing is a complex and challenging task. It requires appreciation of the underpinning pharmacology combined with numerous practical competencies that ensures safe translation into clinical practice. Various studies suggest confidence and competence in prescribing can be increased through the use of targeted education programmes. [5–9] Unfortunately, medical schools have little evidence to support how best to prepare students for their lives as prescribers [10].

At the University of Birmingham, final-year medical students’ knowledge and skills in therapeutics have been assessed in the past with an objective structured clinical examination (OSCE) of prescribing skills [11].

Based on student feedback, it was felt that a reform of the curriculum and assessment was required to ensure that it was relevant to the skills and knowledge required of newly qualified doctors. We therefore undertook a service evaluation with the aim of ascertaining overall satisfaction with undergraduate teaching, identifying which practical or counselling tasks, relating to therapeutics, were frequently being performed and to highlight areas of initial as well as persistent difficulties for PG1 doctors.

## Methods

### Study design

Retrospective questionnaire-based service evaluation. All participants provided consent before enrolling. Ethical approval from the UK national ethics service was not sought as this work was a service evaluation of the therapeutics teaching offered by the University of Birmingham’s School of Medicine.

### Participants

PG1 doctors working at eight teaching hospitals in the Birmingham and Worcester regions between July and August of 2010 participated. At the time of questionnaire distribution participants were nearing the end of, or had recently completed their PG1 training. Questionnaires were anonymous and completed voluntarily.

### Data collection and statistical analysis

The questionnaire was initially piloted on 36 PG1 doctors (presented at the British Pharmacological Society Winter Meeting, December 2008) to identify sources of misinterpretation and to ensure acceptability. These 36 responses were not included in the analysis.

The questionnaire was initially distributed at Queen Elizabeth Hospital, Selly Oak Hospital, and Birmingham City Hospital until 88 participants were enrolled. The questionnaire was then revised to include two additional questions, focusing on prescribing in special groups, and on medicines PG1s were comfortable to prescribe at the end of their first year.

The final 25-point questionnaire included questions on demographics, satisfaction with undergraduate therapeutics training, prescribing difficulties encountered, frequency of undertaking various practical or counselling tasks related to therapeutics, confidence in drug usage, and areas they felt the undergraduate pharmacology curriculum could be improved.

Frequency of undertaking practical tasks and frequency of counselling were assessed by asking participants how many times they had undertaken them as a PG1 doctor with answers categorized to 0 times, 1–5 times, 6–10 times, and more than 10 times. To assess satisfaction with undergraduate therapeutics teaching, a 5-point Likert Scale was used, ranging from ‘strongly agree’ to ‘strongly disagree’. Qualitative analysis of free text comments was used to collect feedback on how therapeutics teaching could be improved with the question ‘Based on the requirements of a PG1 doctor, how do you think learning in undergraduate clinical pharmacology/therapeutics could be improved?’ Prescribing difficulties were assessed with the question ‘Which written prescriptions or prescription-related tasks do you feel you had difficulty with?’

Numerical data were analyzed using descriptive statistics and differences between dichotomous variables were assessed using *χ* ^2^ tests. Qualitative statements were reduced to their simple meaning. Popular themes were described in terms of the number of participants who expressed them.

## Results

### Participant demographics

Questionnaires were collected from all eight hospitals in Birmingham, Worcester and Herefordshire. Completed questionnaires were returned by 211 of 277 PG1 doctors employed at these schools, giving a response rate of 76 %. Of the respondents, 64 % had undertaken their undergraduate medical degree at the University of Birmingham, and 3 % at Universities outside of the UK. Sixty-two percent (62 %) of participants were female, which is similar to the average current sex ratio in UK medical schools [12].

A general medical rotation had been undertaken by 54 %, and a general surgical rotation had been undertaken by 80 %. A significant proportion had undertaken specialist rotations including: paediatric medicine (10.4 %); ear nose and throat surgery (9.2 %); intensive therapy unit (8.6 %); anaesthetics (7.4 %); and obstetrics and gynaecology (3.8 %).

### Prescription-related difficulties

Respondents were asked to list which written prescriptions or prescription-related tasks they had encountered difficulty with as a postgraduate. Seventy-one percent of respondents reported at least one area of difficulty, with the prescribing of warfarin (16.6 %), controlled drugs (15.6 %) and insulin sliding scales (11.8 %) being the most frequent (Table 1).

**Table 1:** Numbers reporting prescriptions and prescription-related tasks as difficult during PG1 (*N* = 211)

Prescribing difficulty	Number (%)
Warfarin	35 (16.6)
Controlled drugs	33 (15.6)
Insulin sliding scale	25 (11.8)
Medications administered via syringe driver	25 (11.8)
Dosing of medications	22 (10.4)
Heparin infusions	20 (9.5)
Medications administered as continuous infusions	17 (8.1)
Medications with reducing regimens	12 (5.7)
Antibiotics	10 (4.7)
Medications for discharge	10 (4.7)

Only the ten most frequently reported tasks are displayed

### Practical tasks related to prescribing

**Fig. 1:**
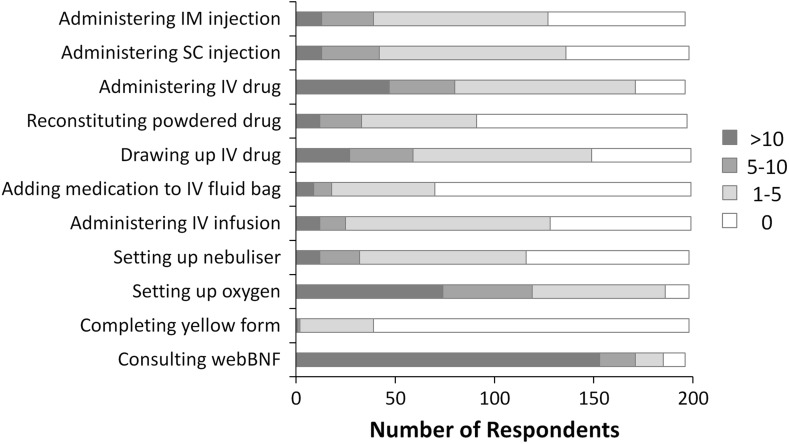
Frequency of undertaking practical prescription related tasks during PG1 (*N* = 211) *IM* intramuscular; *SC* subcutaneous; *IV* intravenous; *webBNF* web-based British National Formulary. *Legend* refers to number of times each task was undertaken during PG1

To determine the day-to-day skills required of PG1 doctors, participants were asked how many times they had been required to undertake various practical therapeutics and prescription related tasks (Fig. 1).

Seventy-five percent of PG1 doctors had prepared a drug for intravenous (IV) administration, and 85.7 % had administered an IV drug. The drugs most commonly prepared were opiates (15.6 %), antibiotics (12.8 %), anaesthetic induction agents (4.7 %) antiemetics (4.3 %), tetracosactrin (Synacthen™) (3.8 %), calcium gluconate (3.3 %) and naloxone (3.3 %). Intramuscular (IM) and subcutaneous injections had been administered by 64.5 % and 67.3 % of participants, respectively. The commonest IM medication to be administered was tetracosactrin (Synacthen™) (13.7 %), followed by vaccinations (9.0 %) and sedatives (2.4 %).

Only 57 % had ever set up a nebulizer. Nearly all had set up and administered oxygen therapy (93 %) with over a third (36 %) having done this on more than ten separate occasions. The least commonly performed task was completing a ‘yellow card’ to report an adverse drug reaction.

### Prescribing to special groups

**Fig. 2:**
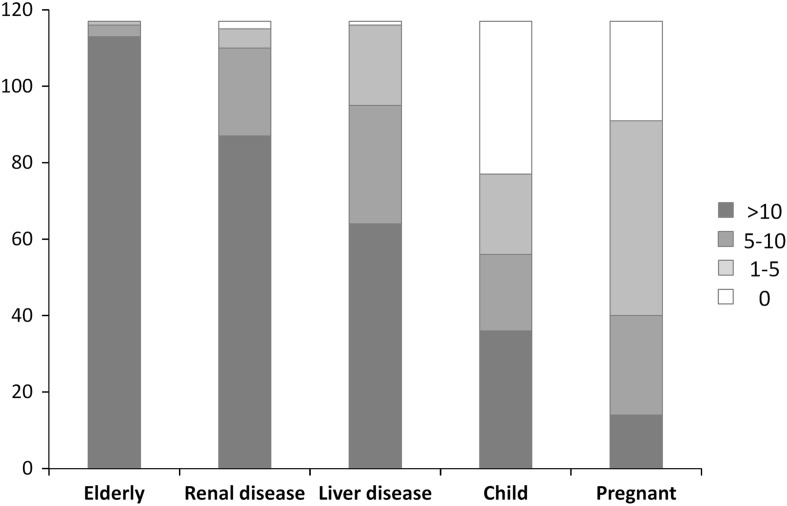
Frequency of prescribing to specialist groups during PG1 (*N* = 123) *Legend* refers to number of times each task was undertaken during PG1

All respondents reported prescribing to special patient groups during their PG1 years. All had prescribed to an elderly patient, and 97 % reported doing so on more than ten occasions. Nearly all had been required to prescribe to a patient with renal disease (98 %) or liver disease (99 %). Most (66 %) had prescribed to children, and 78 % had prescribed to pregnant women (Fig. 2). There was a significant relationship between those that had undertaken placements in specialist areas and the frequency with which they had been required to prescribe to that group. PG1 doctors who had been attached to paediatrics (*p* < 0.005) or obstetrics/gynaecology (*p* = 0.003) more frequently prescribed to these groups.

### Counselling

Almost all PG1 doctors (96 %) had been required to counsel patients regarding warfarin usage, 46 % doing so on a frequent basis (more than 10 times). A minority had frequently counselled on the use of methotrexate (1.5 %) or bisphosphonates (4.4 %) (Table 2).

**Table 2:** Percentage frequency of those counselling patients for medications and medication related devices (*N* = 211)

Medication/device	Frequency of counselling (%) [*N* = 211]		
	Never	Infrequently	Frequently
Warfarin	4.4	49.0	46.6
Antibiotics	13.5	35.8	50.7
Inhalers	20.9	43.1	35.4
Steroids	24.5	46.4	29.1
Aspirin	20.5	51.2	28.3
Antihypertensives	26.7	46.1	27.2
Peak flow meter	22.3	52.4	25.2
Insulin	27.1	48.3	24.6
Nebulizer use	35.3	40.1	24.6
GTN spray	19.5	56.6	23.9
Statins	31.7	46.8	21.5
Bisphosphonates	55.8	39.8	4.4
Methotrexate	82.0	16.5	1.5

*Never* 0 times during PG1, *Infrequently* 1–9 times during PG1, *Frequently* greater than 10 times during PG1

### Satisfaction with undergraduate training

Sixty-three percent agreed that their undergraduate training had equipped them with the knowledge and skills they required as an PG1 doctor, and 7 % strongly agreed with this. Twenty percent disagreed or strongly disagreed that their undergraduate training in pharmacology had been adequate. The remainder were undecided (14 %) or failed to answer the question (3 %). No significant difference was found between local graduates and graduates from other UK medical schools (*p* = 0.52).

### Areas for improvement in undergraduate teaching

Written feedback was provided by 105 participants. These responses were reduced to nine themes. The most popular suggestion was for teaching to be made more relevant to clinical practice which was a theme expressed by 56.2 % of respondents.

Other suggested areas for improving the therapeutics teaching at undergraduate level included more practice at prescription writing (16.2 % of those that responded), teaching with clinical examples (14.3 %), more practical procedures training (13.3 %) and more therapeutics teaching overall (10.5 %).

### Comfortable to prescribe without supervision

**Fig. 3:**
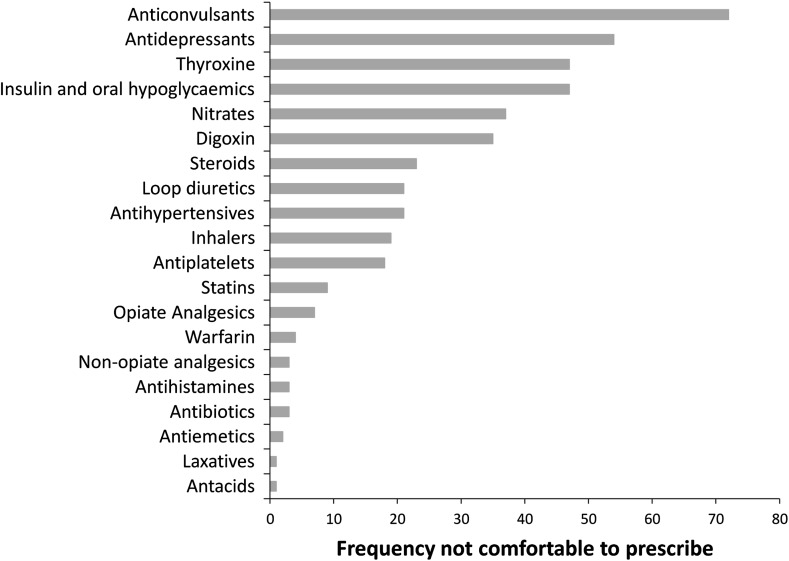
Number not comfortable to prescribe medications at the end of PG1 (*N* = 123)

To assess continued development during their first postgraduate year, doctors were asked which medications they now felt comfortable to prescribe without supervision. The majority were comfortable to prescribe antacids, laxatives, antiemetics, antibiotics, antihistamines, non-opiate analgesia and warfarin. Most were not comfortable to prescribe anticonvulsants, antidepressants, thyroxine and hypoglycaemics (insulin or oral) (Fig. 3).

## Discussion

This is one of the first studies to describe the patterns of therapeutic tasks undertaken by PG1 doctors in day-to-day clinical practice. Their confidence to undertake these tasks has been described, and feedback on their undergraduate therapeutics teaching has been collected. The response rate across the Birmingham regions was high at 76 %, minimizing responder bias.

On commencing PG1, a large proportion expressed difficulty in prescribing warfarin and controlled drugs. These drugs are commonly prescribed by junior doctors, and are potentially the most dangerous if errors are made in prescribing them. It would therefore seem appropriate for undergraduate training to more extensively cover the safe prescription of these medications. Participants also described lower confidence in prescribing syringe-driven and infused drugs, in particular, heparin and insulin. It should be noted that such regimens are often prescribed in accordance to hospital-specific guidelines and it may therefore be more appropriate for each hospital to provide adequate training in such areas during initial orientation. While questionnaires were anonymous, it should be noted that descriptions of therapeutic difficulties might have been influenced by acceptability bias with a tendency for participants to underplay personal weaknesses. Further, for specific medications with which problems were reported, the exact area of difficulty encountered requires further delineation and is an area for future research.

A high proportion of participants reported commonly setting up and administering oxygen therapy, as well as administering intravenous drugs. Both IV administration and oxygen administration are often undertaken in an acute or emergency setting and require a practical understanding of the available equipment. Reports of confidence to set-up oxygen were high in a previous study [13]. Nevertheless, it may be appropriate for medical schools to ensure that all students have undertaken a form of practical training and assessment.

A previous study has shown only 15 % of PG1s to be confident in administering IV drugs [5]. In the current study, the intravenous drugs which participants reported giving most frequently were morphine and antibiotics. It may therefore be appropriate for undergraduate training to include practical sessions in administering these. Teaching should emphasize certain points such as checking for allergy before administration, a step that has been overlooked by junior doctors in previous research [14]. Few respondents were required to set up or administer intravenous infusions or nebulizers suggesting that it may be appropriate to spend less time on teaching these tasks during undergraduate training.

Nearly all participants reported prescribing to the elderly and those with renal and liver disease. Fewer prescribed to pregnant women or children, although as PG1s at the region’s children’s and women’s hospitals were not included, this may have been influenced by selection bias. Medical schools should therefore ensure undergraduate training includes the principles of safely prescribing in special groups, with particular focus on renal, liver and elderly patients.

The majority of participants reported frequently using the webBNF. This relatively new resource may be utilized in place of the printed British National Formulary (BNR) which has been heavily relied on in the past [13]. Future undergraduate training should therefore equip students to use this resource safely.

Almost all participants reported completing a yellow card for suspected adverse drug reaction 0–5 times. This low usage may be due to lack of confidence or experience, and medical schools may consider providing specific training on when and how this should be completed.

Educating patients about their medications has been shown to improve patient understanding, increase compliance and reduce attendance at primary care and admissions to secondary care [15]. Our study demonstrates that participants frequently counselled patients starting on antibiotics, warfarin, and inhalers. In a previous study, only 30 % of PG1s expressed confidence in providing such information [13]. Undergraduate teaching and assessment of these common counselling scenarios may improve low confidence in this area.

Only 65.2 % of participants felt their undergraduate therapeutics/pharmacology training had adequately equipped them for their PG1 duties. This proportion was similar in Birmingham and non-Birmingham graduates suggesting that this finding may be generalizable across UK medical graduates, although numbers of non-Birmingham graduates were small. In a nationwide survey of 2413 UK medical students, 74 % felt their training was inadequate to meet the General Medical Council prescribing competency requirements. It should be noted that the University of Birmingham has a distinct course and assessment in pharmacology and therapeutics, which most other Universities lack [5]. Heaton demonstrated that distinct assessments were associated with increased satisfaction which may explain why our study demonstrates a lower level of dissatisfaction than the national average [5]. We did not ask participants about satisfaction with other clinical areas. It might therefore be argued that the dissatisfaction and uncertainty expressed with therapeutics merely reflects the general uncertainty of a newly qualified doctor. Contrary to this view a study in Plymouth, UK, suggested that the degree of confidence and satisfaction of PG1s in a variety of tasks, not only prescribing, is related to the undergraduate curriculum design [16].

A strong message from participant’s feedback was for teaching to be made more relevant to clinical practice. Further suggestions were for more training on prescription writing, more training using clinical examples and more practical sessions. These findings are supported by previous research with one study finding only 38 % of PG1s to be confident to write prescriptions and only 35 % to have filled in a hospital prescription chart more than three times before graduating [5]. Participants also suggested more therapeutics training overall. Significant improvements in confidence and reductions in prescription errors have been demonstrated with increased therapeutics and pharmacology undergraduate training [17].

At the end of PG1, confidence improved, although there were some medicines participants were still not happy to prescribe. These included anticonvulsants, antidepressants, insulin, oral hypoglycaemics, thyroxine, nitrates and digoxin. It may be more appropriate for postgraduate training to provide teaching on these areas of difficulty which appear to persist to the end of PG1.

## Conclusion

There has been limited research into the prescribing patterns and prescribing confidence of junior doctors in the UK. In this survey, many expressed difficulties with prescribing potentially dangerous medications such as warfarin, controlled drugs and syringe-driven drugs. Undergraduate training must equip graduates to be able to safely prescribe these. Although better than previous UK-based surveys, a significant proportion were unsatisfied with undergraduate therapeutics teaching. Feedback produced the strong suggestion for undergraduate teaching to emphasize topics relevant to clinical practice. This survey has highlighted the therapeutics-related tasks that PG1s commonly and uncommonly undertake and such information is essential for guiding undergraduate teaching. Further research to describe the therapeutic responsibilities of PG1s, as well as the quality of undergraduate training, is required to validate these findings and to further improve safety in prescribing.

## Essentials

- Many junior doctors expressed difficulties in prescribing potentially dangerous medications.
- There was a strong opinion for undergraduate teaching to become more practical and more relevant
- We report on the common practical prescribing tasks undertaken by newly qualified doctors. This can guide undergraduate curricula and better prepare the student for prescribing life
- At the end of their first postgraduate year prescribing confidence had improved but deficiencies remained. These areas have been described and should be targeted in postgraduate training.
